# Systematic review and network meta-analysis of the effects of plant active substance on quality of life in breast cancer patients

**DOI:** 10.3389/fphar.2025.1622479

**Published:** 2025-11-28

**Authors:** Lu Wang, Xiaofan Xu, Mengmeng Guo, Yanxu Dong, Helin Ding, Shuai Hao, Liqun Huo, Yuqing Song, Jun Gu, Zhenghong Yu

**Affiliations:** 1 Jinling Clinical Medical College, Nanjing University of Chinese Medicine, Nanjing, China; 2 Department of General Surgery, Jinling Hospital, Medical School of Nanjing University, Nanjing, China; 3 Department of Breast Surgery, Nantong Maternal and Child Healthcare, Nantong, China

**Keywords:** breast cancer, plant active substance, network meta-analysis, quality of life, randomized controlled trials

## Abstract

**Background:**

Plant-derived active substances are increasingly recognized as potential adjuvant therapies in breast cancer treatment, with emerging evidence suggesting their positive impact on both treatment outcomes and quality of life (QoL). This study aims to evaluate the effects of fifteen active substances—soy capsules, Helixor A, *Viscum album* [L.] extracts, *Withania somnifera*, *Paullinia cupana*, *P. ovata* husk, ginseng, curcumin, Jollab, ginger, fermented soybean extract, mistletoe extract, robuvit®, peppermint extract, and Chlorella extract—on QoL in patients with breast cancer (BC).

**Methods:**

A comprehensive literature search was performed across PubMed, EMBASE, Cochrane Library, and Web of Science up to January 2025. The primary outcomes of interest included QoL, fatigue, pain, physical functioning, nausea, and role and emotional functioning. Statistical analyses were carried out using StataMP 15.1 software. Treatment efficacy was assessed using Surface Under the Cumulative Ranking Curve (SUCRA) probabilities. Additionally, cluster analysis was conducted to examine the multidimensional effects of natural extracts across these seven clinical outcomes.

**Results:**

After screening, 18 eligible studies were included, encompassing 2062 patients and evaluating 15 substances. The analysis incorporated patient-reported outcomes from multiple trials: QoL (11 studies), fatigue (10 studies), pain (8 studies), physical functioning (8 studies), nausea (7 studies), role functioning (7 studies), and emotional functioning (7 studies). Based on SUCRA values, *Withania somnifera* was identified as the most effective treatment for enhancing QoL (99.4%) (SMD = 4.66, 95% CI: 3.47–5.85), physical functioning (100.0%) (SMD = 7.78, 95% CI: 6.61–8.96), role functioning (100.0%) (SMD = 8.10, 95% CI: 6.89–9.32), and emotional functioning (100.0%) (SMD = 5.71, 95% CI: 4.81 to 6.61) were more effective than the standard treatment. Moreover, *Withania somnifera* was found to be the most promising option for reducing fatigue (99.9%), pain (100.0%), and nausea (98.6%).

**Conclusion:**

The network meta-analysis indicates that *Withania somnifera* was effective in enhancing quality of life and contributed to a reduction in therapeutic side effects in BC patients. Our findings support the therapeutic potential of plant bioactive substances in breast cancer care; however, further clinical validation of their efficacy is warranted.

**Systematic Review Registration:**

https://www.crd.york.ac.uk/prospero/, identifier CRD420251006422.

## Introduction

Breast cancer (BC) is one of the most prevalent malignant cancers affecting women globally. The risk factors for BC include, but are not limited to, hormonal status, ionizing radiation, obesity, and genetic factors (Smolarz, Nowak, and Romanowicz, 2022; Fan et al., 2014). According to statistical data from 2022, the global incidence of new breast cancer cases was estimated at approximately 2.309 million, making it the second most common cancer. Additionally, around 666,000 global breast cancer deaths placed it fourth in mortality rates, posing a substantial risk to the health of women worldwide (Bray et al., 2024). Common treatment modalities for BC include surgery, chemotherapy, radiotherapy, and hormone therapy. In recent years, targeted therapy and immunotherapy have contributed to longer survival rates for patients. However, patients undergoing these treatments may experience a high risk of adverse effects, including pain, cardiovascular disease, and diarrhea (Strijbos et al., 2024; García-Aranda and Redondo, 2019; Jerusalem, Lancellotti, and Kim, 2019), leading them to explore adjuvant therapeutic methods aimed at improving quality of life and reducing side effects. The application of plant bioactive substances in the treatment of breast cancer is increasingly gaining attention (Liang et al., 2019; Wang et al., 2020).

Plant bioactive substances can be derived from a variety of natural plants, including Green Tea, Red Clover, Boswellia serrata, and Cimicifuga racemosa, through advanced techniques such as ultrasound-assisted extraction, decoction extraction, and solvent extraction (Dostal et al., 2015; Ferraris et al., 2020; Wang et al., 2019; Valente et al., 2024). The therapeutic role of plant bioactive substances in breast cancer (BC) management has gained increasing prominence, primarily due to their ability to enhance treatment efficacy while mitigating the toxic side effects associated with conventional therapies in breast cancer patients (Wang et al., 2022; Valente et al., 2024). Research by J. Chen et al. demonstrated that Salvia miltiorrhiza effectively mitigated skin flap ischemia and prevented necrosis following mastectomy procedures (Chen et al., 2010). Furthermore, a study by Hamidian and colleagues revealed that prophylactic administration of ginseng supplementation may provide cardioprotective benefits against doxorubicin-induced early-onset cancer therapeutics-related cardiac dysfunction (CTRCD) and mitigate the decline in left ventricular ejection fraction (LVEF) in breast cancer patients undergoing treatment (Hamidian et al., 2023a). Additionally, research has suggested that isopropanolic black cohosh extract may prolong disease-free survival in breast cancer patients (Henneicke-von Zepelin et al., 2007).

However, most studies focus on the effects of individual plant bioactive substances on breast cancer (BC) patients without directly comparing different plant extracts (Brooker et al., 2006; Khosropanah et al., 2023; Kedzierska et al., 2012). The outcomes of various clinical studies exhibit significant heterogeneity, and there is a limited number of evidence-based medicine studies available in this area (Chung et al., 2015). Therefore, determining the optimal therapeutic approach within complex treatment regimens for BC patients is a critical clinical priority. Network Meta-Analysis (NMA) is an advanced statistical method that simultaneously compares the effects of multiple interventions, integrating both direct and indirect evidence to quantitatively evaluate and rank interventions within a complex treatment network (Chang et al., 2022; Shehab et al., 2025). In this article, we will use network meta-analysis to compare the therapeutic effects of different plant active substance in breast cancer patients, in order to provide effective evidence-based support for clinical decision-making.

## Methods

This meta-analysis was rigorously conducted in accordance with the Systematic Reviews and Meta-Analyses (PRISMA) guidelines. The research protocol has been formally registered with the PROSPERO registry under ID: CRD420251006422.

### Search strategy

Investigators conducted a comprehensive search across four major electronic databases (PubMed, EMBASE, the Cochrane Central Register of Controlled Trials, and Web of Science), spanning from the inception of each database until January 2025. Additionally, a manual search was performed by including references from the relevant literature. The search strategy was designed around intervention methods, utilizing specific search terms such as Breast Neoplasms, Plant Active Substances, Luteolin, Apigenin, Icariin, Gallic Acid, Berberine, Ginsenoside, Paeoniflorin, and Astragalus Polysaccharide. The detailed search methodologies are outlined in Table 1, with PubMed serving as an example. The process of study selection, data extraction, and evaluation of the risk of bias was rigorously carried out by two independent researchers.

**TABLE 1:** Search strategy on PubMed.

Search	Search strategy on PubMed
#1	(Breast Neoplasms [MeSH Terms]) OR (Triple Negative Breast Neoplasms [MeSH Terms])
#2	((((((((((((((((((((((((((((((((((((((((((((((((((((Breast Neoplasms [Title/Abstract]) OR (Triple Negative Breast Neoplasms [Title/Abstract])) OR (Breast Neoplasm [Title/Abstract])) OR (Neoplasm, Breast [Title/Abstract])) OR (Neoplasms, Breast [Title/Abstract])) OR (Breast Tumors [Title/Abstract])) OR (Breast Tumor [Title/Abstract])) OR (Tumor, Breast [Title/Abstract])) OR (Tumors, Breast [Title/Abstract])) OR (Breast Cancer [Title/Abstract])) OR (Cancer, Breast [Title/Abstract])) OR (Cancer of Breast [Title/Abstract])) OR (Cancer of the Breast [Title/Abstract])) OR (Malignant Neoplasm of Breast [Title/Abstract])) OR (Breast Malignant Neoplasm [Title/Abstract])) OR (Breast Malignant Neoplasms [Title/Abstract])) OR (Malignant Tumor of Breast [Title/Abstract])) OR (Breast Malignant Tumor [Title/Abstract])) OR (Breast Malignant Tumors [Title/Abstract])) OR (Mammary Cancer [Title/Abstract])) OR (Cancer, Mammary [Title/Abstract])) OR (Cancers, Mammary [Title/Abstract])) OR (Mammary Cancers [Title/Abstract])) OR (Mammary Neoplasms, Human [Title/Abstract])) OR (Human Mammary Neoplasm [Title/Abstract])) OR (Human Mammary Neoplasms [Title/Abstract])) OR (Neoplasm, Human Mammary [Title/Abstract])) OR (Neoplasms, Human Mammary [Title/Abstract])) OR (Mammary Neoplasm, Human [Title/Abstract])) OR (Breast Carcinoma [Title/Abstract])) OR (Breast Carcinomas [Title/Abstract])) OR (Carcinoma, Breast [Title/Abstract])) OR (Carcinomas, Breast [Title/Abstract])) OR (Mammary Carcinoma, Human [Title/Abstract])) OR (Carcinoma, Human Mammary [Title/Abstract])) OR (Carcinomas, Human Mammary [Title/Abstract])) OR (Human Mammary Carcinomas [Title/Abstract])) OR (Mammary Carcinomas, Human [Title/Abstract])) OR (Human Mammary Carcinoma [Title/Abstract])) OR (ER-Negative PR-Negative HER2-Negative Breast Cancer [Title/Abstract])) OR (ER Negative PR Negative HER2 Negative Breast Cancer [Title/Abstract])) OR (ER-Negative PR-Negative HER2-Negative Breast Neoplasms [Title/Abstract])) OR (ER Negative PR Negative HER2 Negative Breast Neoplasms [Title/Abstract])) OR (Triple Negative Breast Cancer [Title/Abstract])) OR (Triple-Negative Breast Cancer [Title/Abstract])) OR (Breast Cancers, Triple-Negative [Title/Abstract])) OR (Breast Cancer, Triple-Negative [Title/Abstract])) OR (Triple-Negative Breast Cancers [Title/Abstract])) OR (Triple-Negative Breast Neoplasm [Title/Abstract])) OR (Breast Neoplasms, Triple-Negative [Title/Abstract])) OR (Breast Neoplasm, Triple-Negative [Title/Abstract])) OR (Triple Negative Breast Neoplasm [Title/Abstract])) OR (Triple-Negative Breast Neoplasms [Title/Abstract])
#3	(#1) OR (#2)
#4	Plant Active Substance [MeSH Terms]
#5	(((((((((((((Plant Active Substance [Title/Abstract]) OR (Active Substance, Plant [Title/Abstract])) OR (plant Active Substance [Title/Abstract])) OR (extract, Plant [Title/Abstract])) OR (herbal Medicines [Title/Abstract])) OR (Medicines, Herbal [Title/Abstract])) OR (Luteolin [Title/Abstract])) OR (Apigenin [Title/Abstract])) OR (Icariin [Title/Abstract])) OR (Gallic acid [Title/Abstract])) OR (Berberine [Title/Abstract])) OR (Ginsenoside [Title/Abstract])) OR (paeoniflorin [Title/Abstract])) OR (astragalus polysaccharide [Title/Abstract])
#6	(#4) OR (#5)
#7	(#3) AND (#6)

### Inclusion and exclusion criteria

The following inclusion criteria were applied: (1) All participants were diagnosed with breast cancer (BC) through pathological and histological examination; (2) Studies in which the intervention group received treatment utilizing various plant active substances; (3) A comparison of the intervention measures with inactive controls, including placebos, standard care (such as surgical treatment, radiotherapy, chemotherapy, endocrine therapy, or other therapies), no treatment, or habitual diet.

Exclusion criteria were as follows: (1) Non-randomized controlled trials (RCTs); (2) Non-therapeutic clinical research, conference papers, animal or cell experiments, case studies, or review articles; (3) Literature lacking experimental data; (4) Articles that could not be fully accessed.

## Data extraction and management

Two investigators independently reviewed the titles and abstracts of all documents, systematically excluding those that clearly did not meet the established inclusion criteria. Any discrepancies were resolved through discussion, and in cases of persistent disagreement, a third author was consulted for further evaluation to reach a consensus. The following data were recorded from the included randomized controlled trials (RCTs): (1) the first author; (2) publication year; (3) participant characteristics (e.g., sample size, age); (4) intervention measures; (5) statistical results, including the pooled effect estimates for each comparison across all outcomes.

### Quality assessment

Two independent researchers evaluated the quality of the included studies using the Cochrane Collaboration’s tool (version 5.4.1.0) for assessing the risk of bias. The risk of bias was categorized into seven domains: (1) randomization method; (2) allocation concealment; (3) blinding of both researchers and participants; (4) blinding of outcome assessment; (5) completeness of outcome data; (6) selective reporting; (7) other sources of bias. Discrepancies in opinion among researchers were resolved through deliberative discussion. If necessary, a third investigator joined the discussion to reach a consensus. Trials were categorized into three levels based on the number of components with a potential high risk of bias (ROB): high risk (five or more), moderate risk (three or four), and low risk (two or fewer) (Higgins et al., 2011).

### Statistical analysis

Plant active substances were regarded as the intervention measure, with all variables being continuous and expressed as means with standard deviations (SD) (Li and Chen, 2021). To address heterogeneity in outcome measurement units across studies, continuous variables were analyzed using 95% confidence intervals (CI) and standardized mean differences (SMD). A random-effects model was applied to account for potential variations among the included studies (Jackson, Riley, and White, 2011).

Stata MP 15.1 software was used for the analysis, in accordance with the PRISMA guidelines for network meta-analysis (NMA) (Shamseer et al., 2015). A Bayesian framework, incorporating Markov Chain Monte Carlo (MCMC) simulation, was used for the network meta-analysis (NMA). To evaluate the consistency between direct and indirect comparisons, researchers conducted a node-splitting analysis, with a p-value threshold of >0.05 indicating statistical consistency (Supplemenatry Tables S1-S3) (Salanti, Ades, and Ioannidis, 2011). Network diagrams were generated using Stata software. In this graphical representation, nodes represent distinct intervention groups (including different natural extracts and control groups), while connecting lines indicate direct comparisons between these interventions. The visualization parameters were scaled according to study availability, with both node size and line thickness being proportional to the number of studies for each respective intervention or comparison (Chaimani et al., 2013).

The hierarchy of interventions was quantified using P scores, which serve as the frequentist counterpart to the Bayesian surface under the cumulative ranking curve (SUCRA) values. These scores estimate the probability that a treatment is superior to competing interventions, averaged across all comparisons. Ranging from 0 to 1, a P score of 1 represents the highest probability of being the optimal treatment, while a score of 0 indicates the lowest probability. Although P scores and SUCRA values can be interpreted as percentage estimates of treatment effectiveness or acceptability, their clinical interpretation requires caution, as they do not necessarily reflect clinically significant differences between interventions (Marotta et al., 2020). To assess potential small-study effects and publication bias in the network meta-analysis, we constructed a network funnel plot and evaluated its symmetry through visual inspection (Khera et al., 2016).

## Results

**FIGURE 1:**
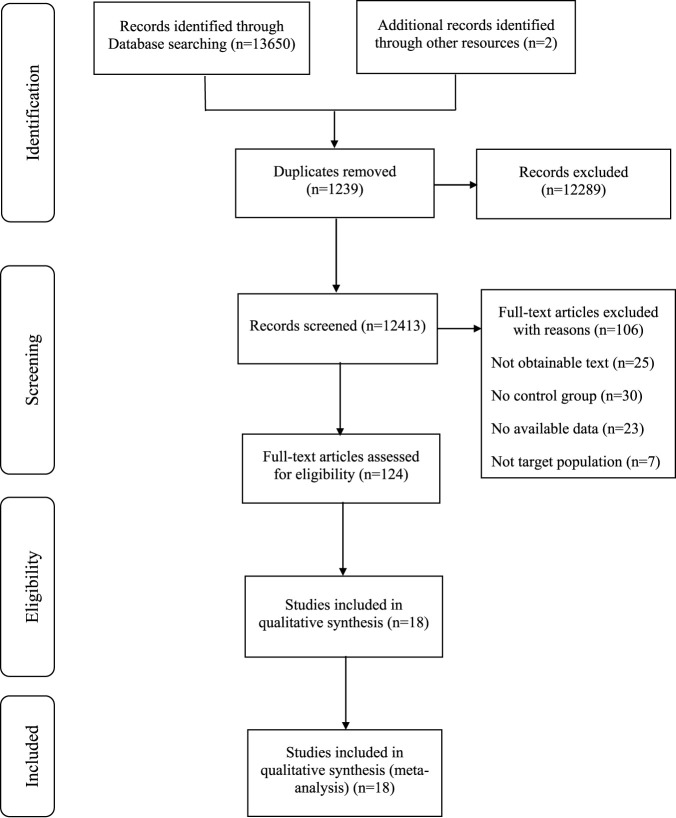
Literature search and screening flow diagram.

A total of 13,650 articles were collected from electronic databases, supplemented by two additional articles obtained through manual searches. Following the removal of 1,239 duplicate articles using a reference management tool, the titles and abstracts of the remaining 12,413 articles were screened, resulting in the exclusion of 112,289 articles. The remaining 124 articles underwent a comprehensive review, resulting in the exclusion of 106 articles for reasons such as being non-randomized controlled trials, containing incomplete data or outcome measures, failing to meet inclusion criteria, or not adhering to the specified interventions of this review. Ultimately, 18 articles met the eligibility criteria and were included in the final analysis of the study (MacGregor et al., 2005; Tröger et al., 2014; Pelzer, Tröger, and Nat, 2018; Biswal et al., 2013; da Costa Miranda et al., 2009; Hasheminasab et al., 2020; Hamidian et al., 2023b; Saghatelyan et al., 2020; Mirzaei et al., 2022; Thamlikitkul et al., 2017; de Oliveira Campos et al., 2011; Chi et al., 2014; Semiglazov et al., 2006; Belcaro et al., 2018; Jafarimanesh et al., 2020; Noguchi, Maruyama, and Yamada, 2014; Semiglasov et al., 2004; Barton et al., 2013) (Figure 1).

**FIGURE 2:**
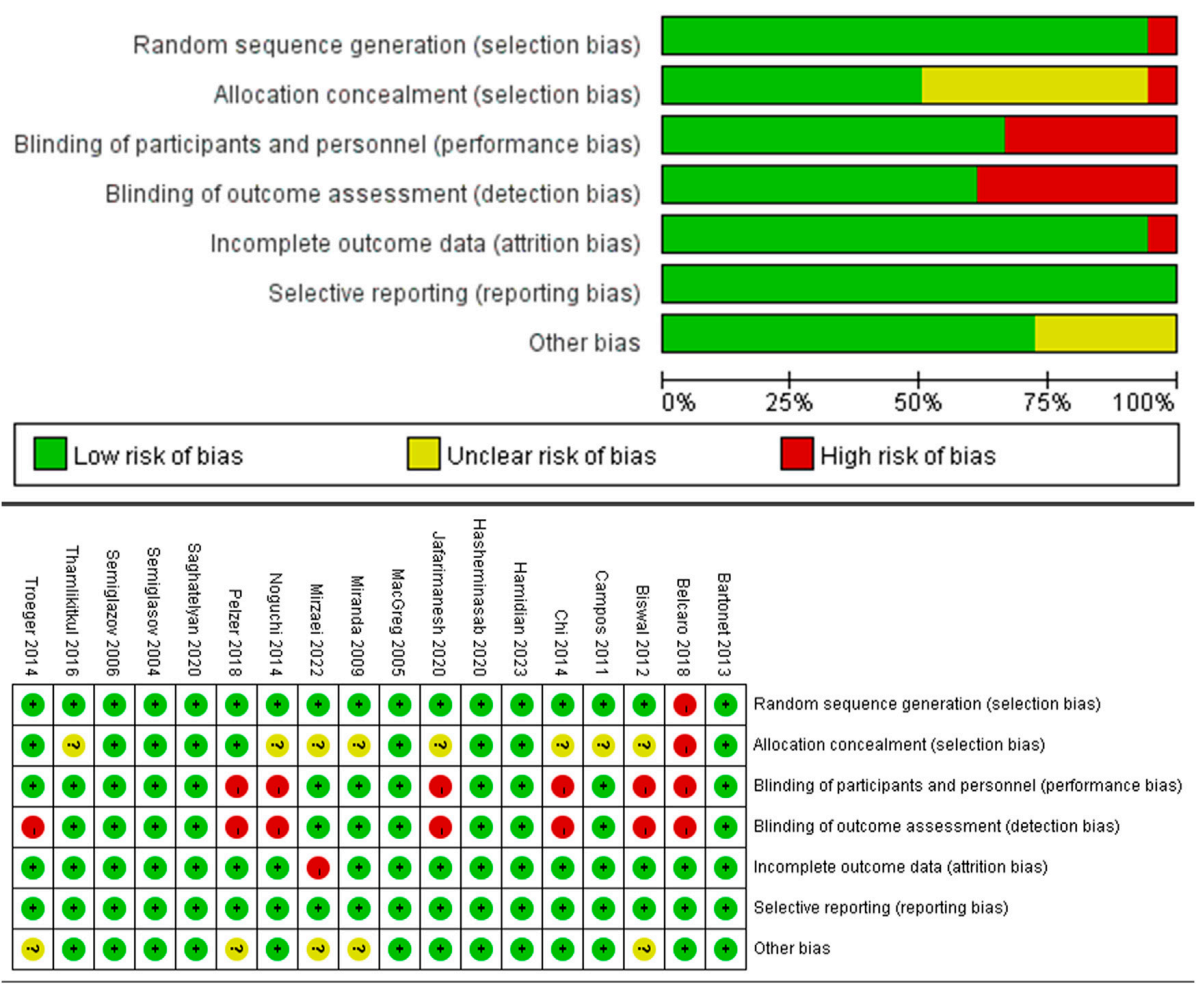
Risk of bias graph for RCT.

All 18 articles included in the analysis were randomized controlled trials (RCTs). Among these, 10 were rated as having a low risk of bias, 8 as having a moderate risk, and none as having a high risk, suggesting that the overall quality of the studies was acceptable. Further details are presented in Figure 2, which provides a visual representation of the risk-of-bias assessment for the included RCTs.

This study analyzed 18 randomized controlled trials involving a total of 2,062 breast cancer patients. The control groups received various interventions, including soy capsules, peppermint extract, *Viscum album* [L.] extracts, *Withania somnifera*, Paullinia cupana, P. ovate husk, ginseng, curcumin, and chlorella extract. Among the studies, 11 used quality of life as an outcome measure, 10 assessed fatigue, 8 evaluated pain severity, 8 assessed physical functioning, 7 measured nausea, 7 reported role functioning, and 7 included emotional functioning as outcome measures (Table 2). A forest plot summarizing pooled effect sizes across outcomes to enhance visual interpretation, which has been included in the Supplementary Material (Supplementary Table S4).

**TABLE 2:** Characteristics of included network meta-analysis. Study characteristics included in network meta-analysis.

Author	Country	Year	Study type	Age	Size	Treatment	Control	Outcome
MacGregor et al.	United Kingdom	2005	RCT	T:51 ± 9.25 C:51 ± 8	T:36 C:36	Soy capsules Duration:12 weeks Dose:70 mg/d	Placebo	Constipation, nausea, headache
Troeger et al.	Serbia	2014	RCT	T:50.4 ± 6.9 C:50.8 ± 8.0	T:34 C:31	Helixor A Duration:54 weeks Dose: 3 × 50 mg/week	No intervention	Quality of life
Pelzer et al.	Serbia	2018	RCT	T:50.0 ± 7.3 C:50.8 ± 7.9	T:64 C:31	*Viscum album* [L.] extracts Duration:18 weeks Dose:3 × 50 mg/week	No intervention	Quality of life
Biswal et al.	Malaysia	2012	Non- RCT	T:51.6 ± 1.0 C:51.8 ± 1.0	T:50 C:50	*Withania somnifera* Duration:18 weeks Dose:3 × 2000 mg/d	No intervention	Quality of life
Miranda et al.	Brazil	2009	RCT	T:59 C:57	T:17 C:19	Paullinia cupana duration: 35 days Dose: 75 mg/d	Placebo	Fatigue symptom
Hasheminasab et al.	Iran	2020	RCT	T:44.74 C:44.74	T:19 C:19	P. Ovate husk Duration:14 days Dose: 3 × 500 mg/d	Placebo	Quality of life
Hamidian et al.	Iran	2023	RCT	T:44 ± 5.8 C:46 ± 8.0	T:15 C:25	Ginseng Duration:10 weeks Dose:1 g/d	Placebo	Quality of life
Saghatelyan et al.	Armenia	2020	RCT	T:57 C:57	T:75 C:75	Curcumin Duration:12 weeks Dose: 300 mg/week	Placebo	Quality of life
Mirzaei et al.	Iran	2022	RCT	T:52.1 ± 9.7 C:52.1 ± 9.7	T:38 C:37	Jollab Duration:4 weeks Dose:3 × 20 mL/d	Placebo	Fatigue
Thamlikitkul et al.	Thailand	2016	RCT	T:49 C:49	T:19 C:15	Ginger Duration:5 days Dose:2 × 500 mg/d	Placebo	Nausea score, adverse events
Campos et al.	Brazil	2011	RCT	T:50.2 ± 12 C:51.8 ± 9.7	T:43 C:32	Paullinia cupana Duration:49 days Dose: 2 × 50 mg/d	Placebo	Fatigue
Chi et al.	Taiwan	2014	RCT	T:57.3 ± 11.9 C:55.3 ± 10.8	T:70 C:73	Fermented soybean extract MicrSoy-20 Duration:8 weeks Dose: 8 mL/d	No intervention	Quality of life, adverse events
Semiglazov et al.	Russia, Bulgaria, Ukraine	2006	RCT	T:46.4 ± 5.9 C:45.9 ± 6	T:176 C:176	Standardised Mistletoe extract PS76A2 Duration:24 weeks Dose: 2 × 0.5 mL/week	Placebo	Quality of life
Belcaro et al.	Italy	2018	RCT	T:49.3 ± 5 C:49.3 ± 5	T:32 C:33	robuvit® Duration:2 months Dose:600 mg/d	No intervention	Quality of life
Jafarimanesh et al.	Iran	2020	RCT	T:49.6 ± 11.8 C51.9 ± 9.5	T:42 C:42	Peppermint extract Duration:4 days Dose: 3 × 40 drops/d	Placebo	Nausea
Noguchi et al.	Japan	2014	RCT	T:50.7 ± 11 C:51.2 ± 10.9	T:23 C:13	Chlorella extract Duration:30 days Dose:2,400–6,000 mg/d	Placebo	Quality of life
Semiglasov et al.	Russia, Ukraine, Bulgaria	2004	RCT	T45.3 ± 5.5 C:43.5 ± 6.1	T:201 C:70	Standardised Mistletoe extract PS76A2 Duration:15 weeks Dose: 2 × 0.5 mL/d	Placebo	Quality of life
Bartonet al.	United States of America	2013	RCT	T:55.3 ± 13 C:55.9 ± 11.8	T:171 C:170	Ginseng Duration:8 weeks Dose: 2000 mg/d	Placebo	Cancer-related fatigue

Abbreviations: T, experimental group; C, control group; RCT, randomized controlled trial.

### Network meta-analysis

**FIGURE 3:**
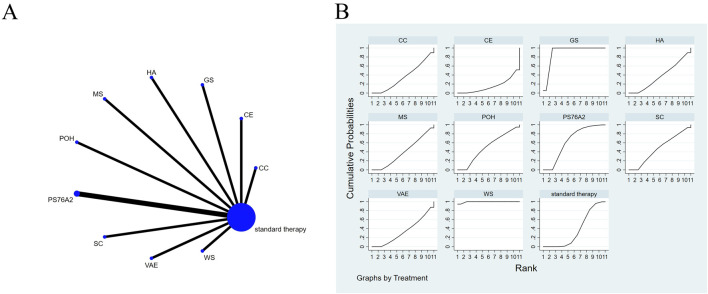
The evidence of plant active substance in influencing quality of life. **(A)**, network graph of the Quality of Life. **(B)**, the SUCRA plot for Quality of Life. Abbreviations: WS, *Withania somnifera*; VAE, *Viscum album* [L.] extracts; SC, soy capsules; PS76A2, an aqueous mistletoe extract standardised to the galactoside-specific mistletoe lectin; POH, P. ovate husk; MS, fermented soybean extract MicrSoy; HA, Helixor A; GS, ginseng; CE, Chlorella extract; CC, curcumin.

**FIGURE 4:**
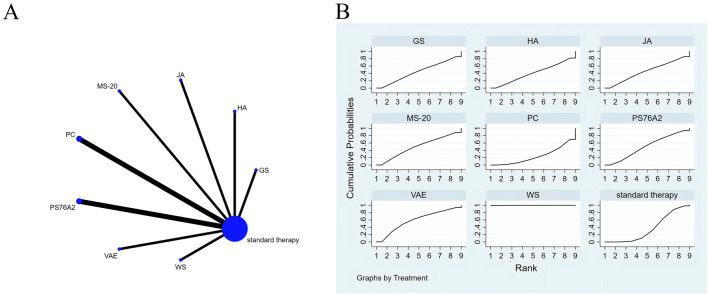
The evidence of plant active substance in influencing fatigue. **(A)**, network graph of the fatigue. **(B)**, the SUCRA plot for fatigue. Abbreviations: WS, *Withania somnifera*; VAE, *Viscum album* fatigue [L.] extracts; PS76A2, an aqueous mistletoe extract standardised to the galactoside-specific mistletoe lectin; PC, *Paullinia cupana*; MS-20, fermented soybean extract MicrSoy20; JA, Jollab; HA, Helixor A; GS, ginseng.

**FIGURE 5:**
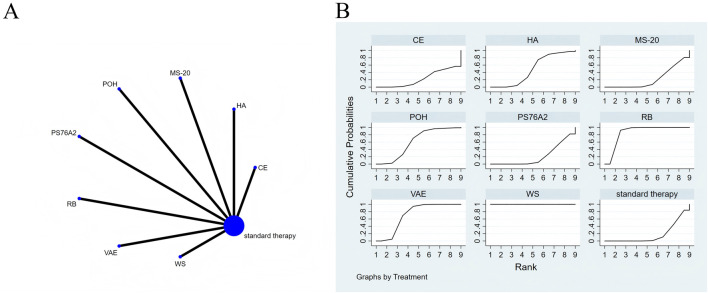
The evidence of plant active substance in influencing pain. **(A)**, network graph of the pain. **(B)**, the SUCRA plot for pain. Abbreviations: WS, *Withania somnifera*; VAE, *Viscum album* [L.] extracts; RB, robuvit® (a natural supplement extracted from the French oak wood); PS76A2, an aqueous mistletoe extract standardised to the galactoside-specific mistletoe lectin; POH, P. ovate husk; MS-20, fermented soybean extract MicrSoy20; HA, Helixor A; CE, Chlorella extract.

**FIGURE 6:**
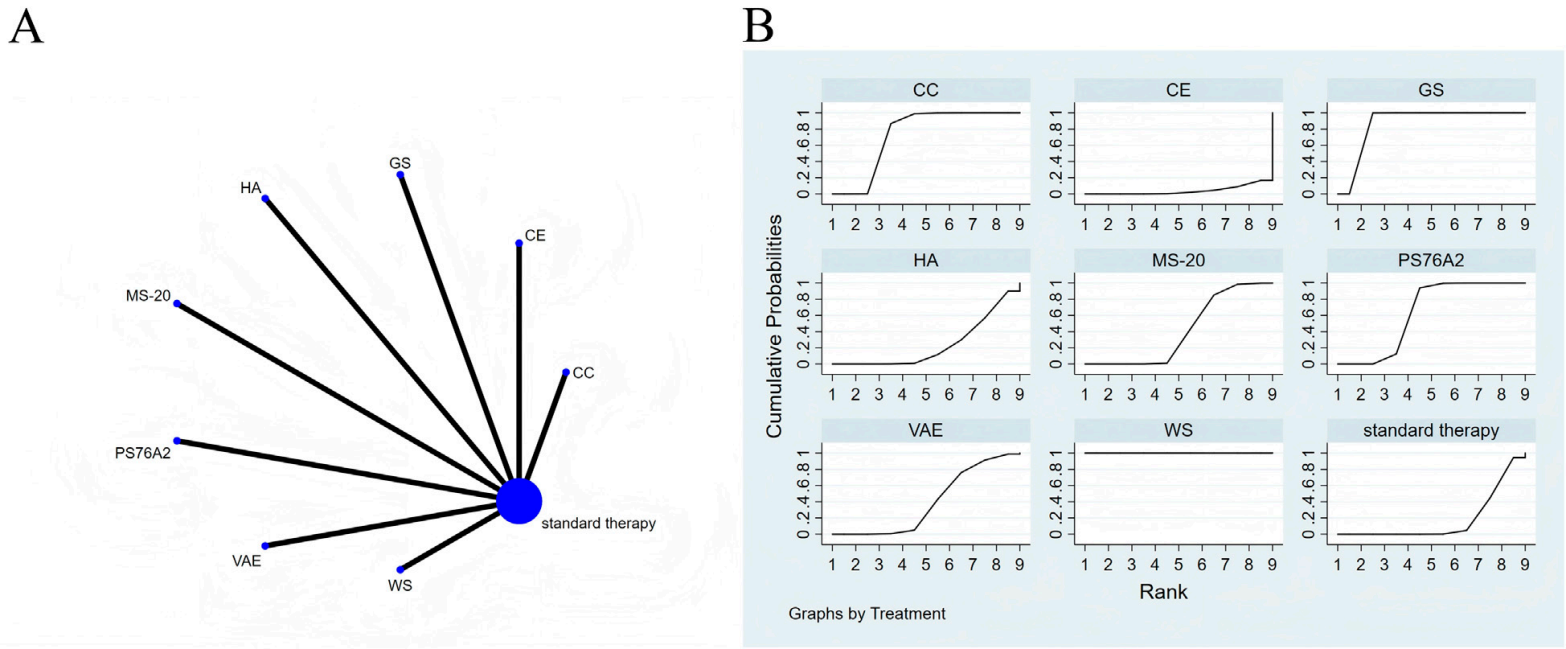
The evidence of plant active substance in influencing physical functioning. **(A)**, network graph of the physical functioning. **(B)**, the SUCRA plot for physical functioning. Abbreviations: WS, *Withania somnifera*; VAE, *Viscum album* [L.] extracts; PS76A2, an aqueous mistletoe extract standardised to the galactoside-specific mistletoe lectin; MS-20, fermented soybean extract MicrSoy20; HA, Helixor A; GS, ginseng; CE, Chlorella extract; CC, curcumin.

**FIGURE 7:**
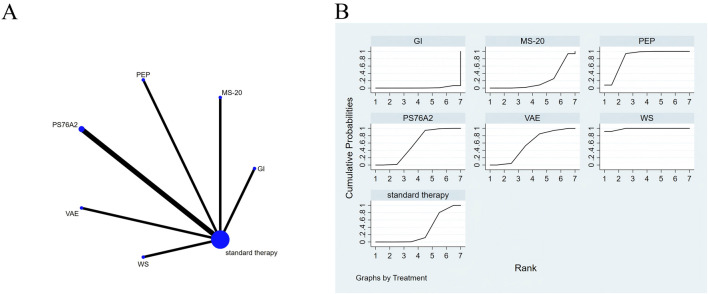
The evidence of plant active substance in influencing nausea. **(A)**, network graph of the nausea. **(B)**, the SUCRA plot for nausea. Abbreviations: WS, *Withania somnifera*; VAE, *Viscum album* [L.] extracts; PS76A2, an aqueous mistletoe extract standardised to the galactoside-specific mistletoe lectin; PEP, Peppermint; MS-20, fermented soybean extract MicrSoy20; GI, ginger.

**FIGURE 8:**
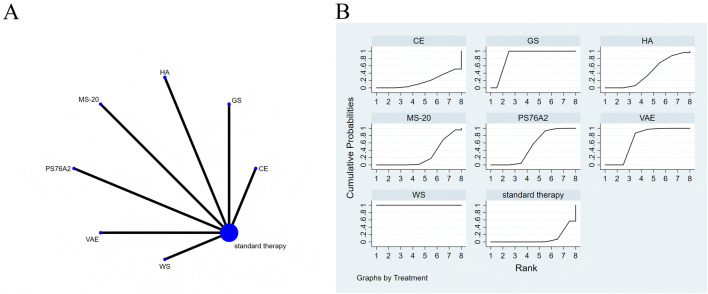
The Evidence of Plant Active Substance in Influencing role functioning. **(A)**, network graph of the role functioning. **(B)**, the SUCRA plot for role functioning. Abbreviations: WS, *Withania somnifera*; VAE, *Viscum album* [L.] extracts; PS76A2, an aqueous mistletoe extract standardised to the galactoside-specific mistletoe lectin; MS-20, fermented soybean extract MicrSoy20; HA, Helixor A; GS, ginseng; CE, Chlorella extract.

**FIGURE 9:**
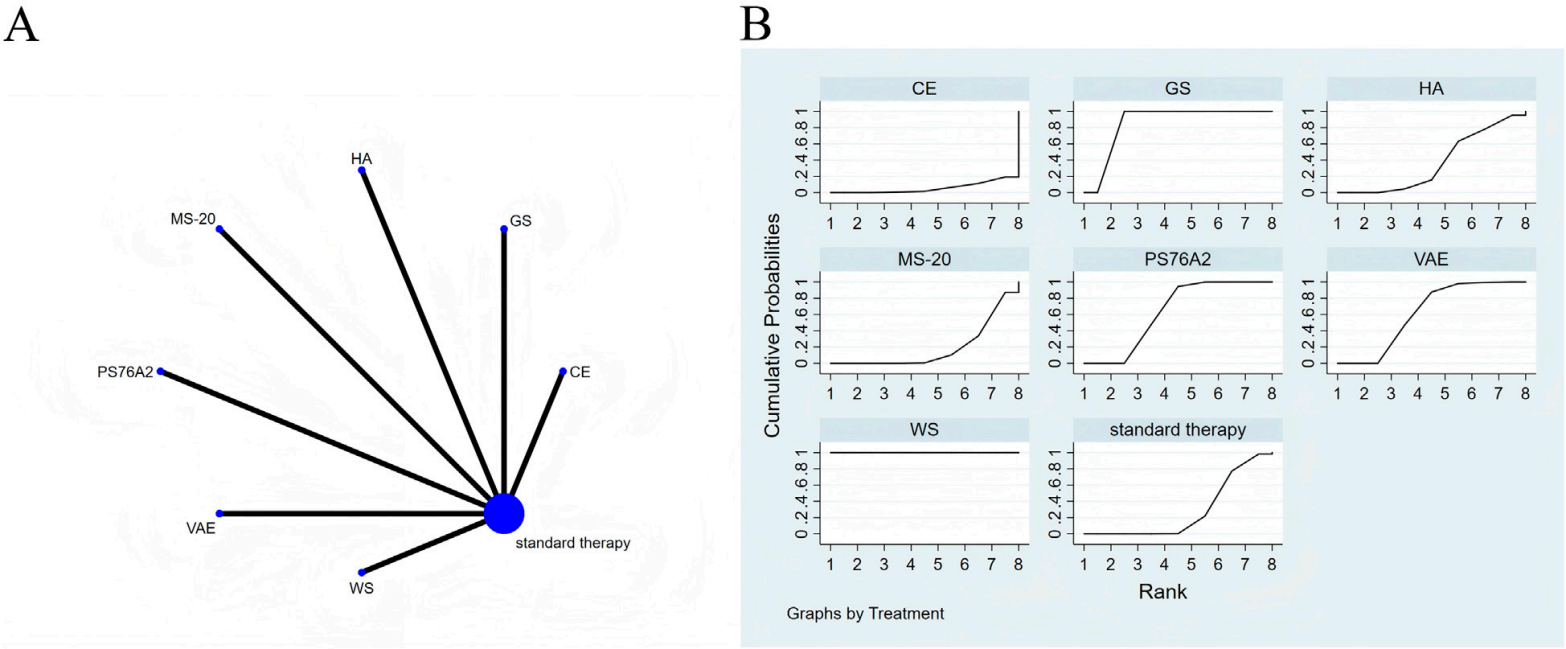
The Evidence of Plant Active Substance in Influencing emotional functioning. **(A)**, network graph of the emotional functioning. **(B)**, the SUCRA plot for emotional functioning. Abbreviations: WS, *Withania somnifera*; VAE, *Viscum album* [L.] extracts; PS76A2, an aqueous mistletoe extract standardised to the galactoside-specific mistletoe lectin; MS-20, fermented soybean extract MicrSoy20; HA, Helixor A; GS, ginseng; CE, Chlorella extract.

The full NMA figure will be shown in Figures 3A, 4A, 5A, 6A, 7A, 8A and 9A.

### Effects of natural extract on quality of life

The results of the network meta-analysis are illustrated in Figure 3A. Consistency and inconsistency tests were performed for all direct and indirect comparisons across the studies, with all p-values exceeding 0.05, indicating that the effect consistency across the studies is satisfactory.

The results of the network NMA indicate that *Withania somnifera* (SMD = 4.66, 95% CI: 3.47–5.85) and ginseng (SMD = 3.16, 95% CI: 1.83–4.50) were more effective than the standard treatment in improving quality of life, with specific details provided in Supplementary Table S5. In the hierarchy of probability for enhancing quality of life through various plant active substances, *Withania somnifera* emerged as the most efficacious, boasting the highest SUCRA value of 99.4%, as depicted in Figure 3B.

### Effects of natural extracts on fatigue

The results of the network meta-analysis are illustrated in Figure 4A. Consistency and inconsistency tests were performed for all direct and indirect comparisons across the studies, with all p-values exceeding 0.05, indicating that the effect consistency across the studies is satisfactory.

The results of the network meta-analysis showed that, relative to the control group’s routine measures, *Withania somnifera* (SMD = −6.12, 95% CI: −8.94 to −3.30) was superior to the control group in reducing fatigue (Supplementary Table S6). In terms of alleviating fatigue, *Withania somnifera* was ranked first in the SUCRA analysis, indicating the highest probability of being the most effective intervention among the evaluated options (SUCRA: 99.9%, as shown in Figure 4B).

### Effects of natural extracts on pain

The network meta-analysis diagram is shown in Figure 5A. All p-values for the indirect and direct comparisons between studies underwent tests for consistency and inconsistency, with all p-values exceeding 0.05, indicating that the consistency of effects among the studies is acceptable.

The results of the network NMA indicate that, compared to standard treatment, *Withania somnifera* (SMD = −6.60, 95% CI: −7.62 to −5.59), robuvit® (SMD = −1.48, 95% CI: −2.04 to −0.93), *Viscum album* [L.] extracts (SMD = −0.91, 95% CI: −1.37 to −0.45), and P. ovate husk (SMD = −0.66, 95% CI: −1.32 to −0.01) were more effective in reducing pain than the control group (Supplementary Table S7). In terms of probability rankings among different plant active substances for reducing pain, *Withania somnifera* ranked first in SUCRA (SUCRA = 100.0%, as shown in Figure 5B).

### Effects of natural extracts on physical functioning

All direct and indirect comparisons across the included studies were assessed for consistency and inconsistency. The resulting p-values were all above 0.05, suggesting that the consistency of effects among the studies was statistically acceptable (Figure 6A).

The NMA results indicate that, in improving physical functioning, *Withania somnifera* (SMD = 7.78, 95% CI: 6.61–8.96), ginseng (SMD = 2.49, 95% CI: 1.62–3.35), curcumin (SMD = 0.99, 95% CI: 0.56–1.42), PS76A2 (SMD = 0.70, 95% CI: 0.48–0.92), and fermented soybean extract MicrSoy20 (SMD = 0.29, 95% CI: 0.02–0.55) were superior to the control group (standard treatment), with details presented in Supplementary Table S8. Regarding probability rankings among different plant active substances for improving physical functioning, St. John’s Wort extract ranked first in SUCRA (SUCRA = 100.0%, as shown in Figure 6B).

### Effects of natural extracts on nausea

Consistency and inconsistency were evaluated for all direct and indirect comparisons across the studies. Since all p-values exceeded 0.05, the consistency of effects across the studies was deemed acceptable (Figure 7A).

The NMA results demonstrated that both *Withania somnifera* (SMD = −1.63, 95% CI: −2.22 to −1.04) and peppermint (SMD = −1.03, 95% CI: −1.62 to −0.44) were significantly more effective in reducing nausea compared to standard treatment, with additional details outlined in Supplementary Table S9. Regarding the probability rankings of plant active substances for reducing nausea, *Withania somnifera* achieved the highest SUCRA value (98.6%), ranking first, as illustrated in Figure 7B.

### Effects of natural extracts on role functioning

Figure 8A presents the network meta-analysis evidence diagram. Tests for consistency and inconsistency were performed on all direct and indirect comparisons, with all p-values exceeding 0.05, indicating that the consistency of effects across the studies was statistically acceptable.

According to the NMA, *Withania somnifera* (SMD = 8.10, 95% CI: 6.89–9.32), ginseng (SMD = 2.68, 95% CI: 1.78–3.57), *Viscum album* [L.] extracts (SMD = 0.91, 95% CI: 0.45–1.37), and PS76A2 (SMD = 0.50, 95% CI: 0.29–0.72) showed superior efficacy in improving role functioning compared to standard treatment (see Supplementary Table S10 for details). In the probability ranking of plant active substances for enhancing role functioning, *Withania somnifera* emerged as the top intervention with a SUCRA value of 100.0%, as depicted in Figure 8B.

### Effects of natural extracts on emotional functioning

The network meta-analysis evidence is presented in Figure 9A. Consistency and inconsistency assessments were conducted for all direct and indirect comparisons across the studies, and all resulting p-values exceeded 0.05, indicating acceptable consistency in the effects among the included studies.

According to the NMA, *Withania somnifera* (SMD = 5.71, 95% CI: 4.81–6.61), ginseng (SMD = 2.70, 95% CI: 1.80–3.60), PS76A2 (SMD = 0.52, 95% CI: 0.30–0.74), and *Viscum album* [L.] extracts (SMD = 0.52, 95% CI: 0.07–0.96) showed superior efficacy in enhancing emotional functioning compared to standard treatment (see Supplementary Table S11 for details). In the probability ranking of plant active substances for improving emotional functioning, *Withania somnifera* emerged as the top intervention with a SUCRA value of 100.0%, as depicted in Figure 9B.

### Publication bias test

**FIGURE 10:**
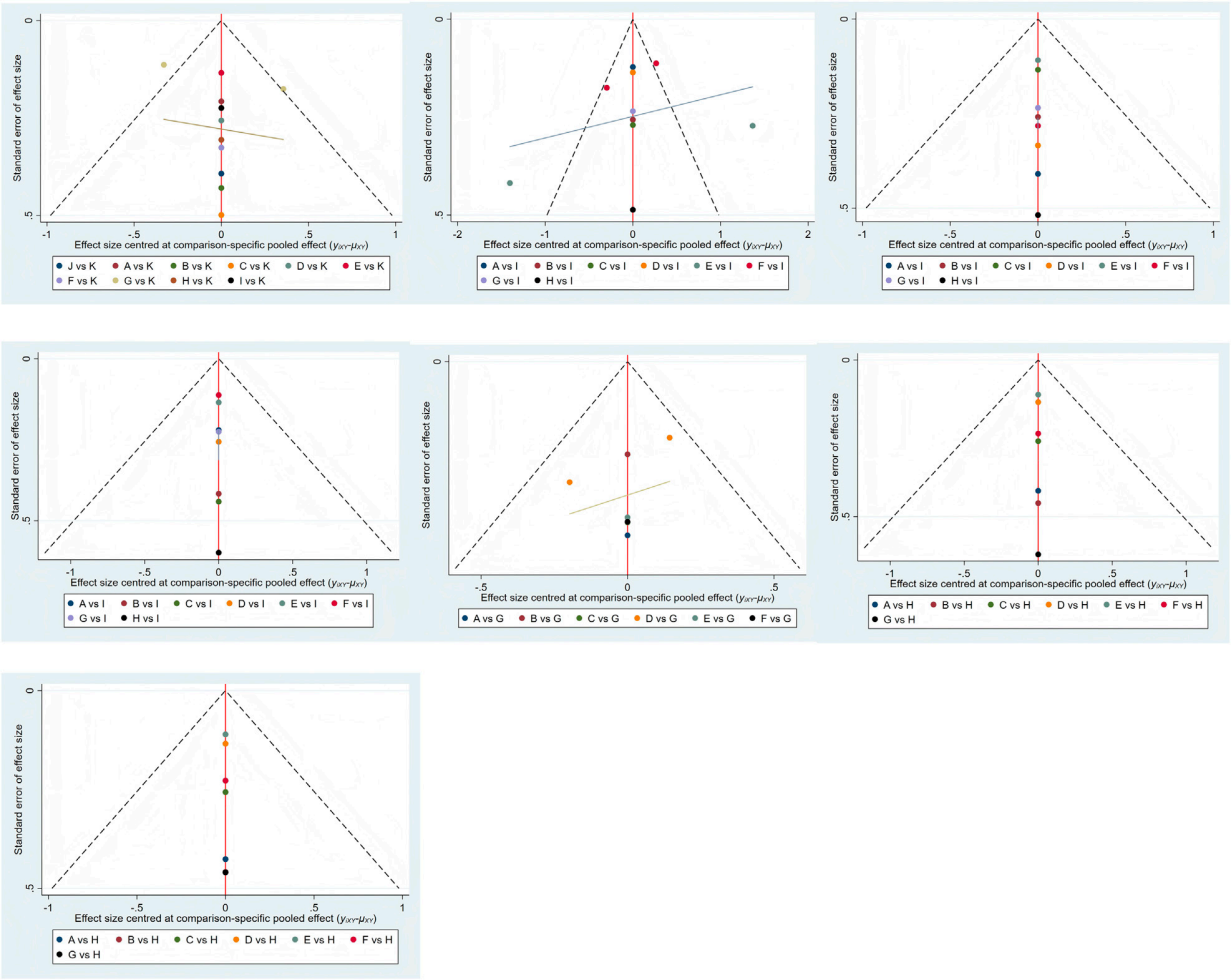
Funnel plot on publication bias.

To assess potential publication bias, individual funnel plots were constructed for each outcome measure. Upon visual examination, the funnel plots did not show substantial evidence of publication bias (Wallace et al., 2009). Further details are provided in Figure 10.

## Discussion

This study evaluated the effects of several natural compounds on overall quality of life, mental health, and adverse reactions in individuals recovering from breast cancer. The analysis included 18 randomized controlled trials involving a substantial cohort of 2,062 breast cancer (BC) patients and examined the effects of fifteen distinct natural substances. The findings indicated that extracts from *Withania somnifera* were effective in enhancing quality of life, while also contributing to reductions in fatigue, pain, and nausea. Furthermore, *Withania somnifera* was associated with improvements in physical functioning, role functioning, and emotional functioning. In conclusion, the study underscores *Withania somnifera* as a beneficial natural option for reducing adverse reactions and improving mental health and overall quality of life in breast cancer patients.

This study demonstrates that *Withania somnifera* offers comparative benefits in enhancing quality of life compared to other plant-based substances. *Withania somnifera* (Indian ginseng or Ashwagandha) is a plant widely used in traditional medicine and is renowned for its diverse biological activities. Its active constituents, such as withaferin A and withanolides, are considered crucial to its pharmacological effects (Singh et al., 2021; Hassannia et al., 2020; Lem et al., 2022). These compounds have been shown to modulate various signaling pathways, including NF-κB, JAK-STAT, and AP-1, thereby reducing cancer-associated inflammation (White et al., 2016; Taniguchi and Karin, 2018). Furthermore, *Withania somnifera* improves reproductive system function by reducing infertility, enhancing spermatogenesis, and promoting sexual behavior in both male and female subjects (Nasimi Doost Azgomi et al., 2018). Studies have reported that *Withania somnifera* (WS) improves semen quality, enhances levels of vitamins E, C, and A, and promotes fertility. These beneficial effects are attributed to WS’s rich composition of alkaloids, ergostane steroids, and essential amino acids, which collectively enhance detoxification processes, reduce oxidative stress, and restore testosterone secretion (Ahmad et al., 2010). Withaferin A has been shown to mitigate oxidative stress by preserving the activity and levels of key antioxidant enzymes, including superoxide dismutase (SOD), glutathione peroxidase (GPx), catalase, and the non-enzymatic antioxidant glutathione (GSH), while reducing thiobarbituric acid reactive species (TBARS). Catalase plays a critical role in cellular homeostasis by decomposing hydrogen peroxide (H_2_O_2_), and its deficiency or dysregulation is implicated in various age-related complication (Nandi et al., 2019). Furthermore, Withaferin A reduced myeloperoxidase (MPO) and nitrotyrosine levels and enhanced Nrf2 signaling in acute pancreatitis, highlighting its antioxidant role. Given that MPO elevation predicts diabetes and cardiovascular risk, MPO suppression could improve outcomes in aging patients (Giovannini et al., 2010).

Breast cancer patients often experience symptoms such as nausea, pain, and fatigue during their treatment (Qi et al., 2024; Zhang et al., 2022). *Withania somnifera* has shown promise in alleviating chemotherapy-induced fatigue and improving the quality of life for breast cancer patients (Biswal et al., 2013). Pro-inflammatory cytokines, such as IL-6 and IFN-γ, contribute to the promotion of neuropathic pain (Koch et al., 2007), whereas the inhibition of these cytokines or the administration of anti-inflammatory cytokines like IL-10 has been shown to alleviate neuropathic pain in animal models (Laumet et al., 2020; Kumar, Sharma, and Garg, 2022). In rat models, treatment with *Withania somnifera* root extract significantly reduced IFN-γ levels while increasing IL-10 expression following SNI-induced neuropathic pain. The analgesic effects of *Withania somnifera* (WS) against both postoperative and neuropathic pain appear to be mediated by its bioactive compounds, particularly withanolides (Lim et al., 2018; Orrù et al., 2014).

Breast cancer significantly impacts the mental and psychological health of patients, particularly during the initial diagnosis and the first year following treatment. Psychological distress is most pronounced during this period (Dinapoli et al., 2021; Fortin et al., 2021). Research indicates that *Withania somnifera* offers a range of potential benefits for mental and psychological health. The root extract of *Withania somnifera*, known as glycowithanolides, has demonstrated anxiolytic and antidepressant effects in rat models, supporting its potential use as a mood stabilizer (Pathak et al., 2021). Studies have found that *Withania somnifera* can be used to promote physical and mental health, restore vitality, and treat various central nervous system disorders, including epilepsy, stress, and neurodegenerative diseases (Kulkarni and Dhir, 2008). Clinical trials suggest that W. somnifera extract enhances cognitive function—including task performance, executive function, attention, and reaction time—in individuals with cognitive impairment, such as older adults with mild cognitive decline and those with schizophrenia, schizoaffective disorder, or bipolar disorder. Additionally, it demonstrates good tolerability, high adherence, and minimal side effects (Ng et al., 2020).

We conducted a statistical analysis of adverse events across the included 18 studies. The findings indicate that the plant extract is both safe and effective. The most frequently reported adverse reaction was injection and infusion site reactions. Other adverse events included mild to moderate nausea, febrile temperature, flatulence and gastritis, all of which resolved without sequelae (Supplementary Table S12).

Overall, our study has clinical implications. *Withania somnifera* has demonstrated superior efficacy in improving the overall quality of life, including physical and functional status, among breast cancer patients, while reducing side effects such as nausea, pain, and fatigue. Additionally, it has shown significant effects in enhancing mental wellbeing. These findings provide valuable insights for breast cancer patients considering plant-based active substances. However, the notably high effect sizes observed for *Withania somnifera* (e.g., SMD >5) warrant careful interpretation. While these estimates suggest a potent intervention effect, their clinical plausibility must be critically evaluated. It is important to consider whether these values may have been influenced by small-study effects, a common source of bias in meta-analyses wherein smaller studies tend to yield larger effect sizes. Future research with larger, rigorously designed trials is needed to validate these findings and clarify the true magnitude of the intervention’s benefit.

### Strengths and limitations

Our study has several notable strengths. It includes 18 research articles involving a substantial cohort of 2,062 patients, providing a robust sample size. The research evaluates 15 distinct natural extracts, adheres to stringent inclusion criteria, systematically identifies all relevant studies that meet predefined conditions, and follows established guidelines for systematic reviews and meta-analyses in its reporting. Additionally, Breast cancer (as well as other forms of cancer) exhibits high incidence and aggressive phenotypes in countries near the equator and in the Southern Hemisphere. We searched regional databases—including SCIELO, ARC, NRF, and AJOL—that focus on geographic areas with high breast cancer prevalence; however, no eligible clinical studies meeting our inclusion criteria were identified.

However, our study also has several inevitable limitations: (1) The prior treatments received by patients undergoing natural extract therapy may influence the evaluation of post-treatment outcomes; (2) There are variations in the doses of natural extracts used across different studies, with a lack of consensus on standardized intervention doses; (3) Variations in the techniques for preparing plant-based active substances and insufficient descriptions of these methods may introduce bias; (4) The impact on patients’ quality of life may also be influenced by factors such as social support systems, which some studies did not consider or report. (5)The inability to perform subgroup analyses due to the limited number of included studies represents a notable limitation of this review, as it restricts the exploration of potential sources of heterogeneity and the examination of differential effects across patient populations or intervention characteristics. (6)The clinical applicability of SUCRA rankings requires cautious interpretation, particularly due to heterogeneity in dosage regimens, pharmaceutical formulations, and treatment durations across included studies.

The conclusion indicates that the effectiveness of plant-based active substances in treating breast cancer is not sufficiently supported by robust clinical evidence, primarily due to the lack of direct comparative data for certain treatments. Consequently, the findings of this study should be interpreted with caution, highlighting the critical need for more comprehensive and detailed research in this area.

## Conclusion

The results of the network meta-analysis (NMA) indicate that *Withania somnifera* is an effective natural product for improving the quality of life, reducing side effects, and promoting mental health in breast cancer patients. Therefore, we recommend the integration of *Withania somnifera* alongside standard treatment to enhance the overall quality of life in these patients. Our study provides valuable insights into the potential of plant-based active substances to improve quality of life, mitigate side effects, and support mental health in breast cancer patients. However, the limitations of the initial study design resulted in indirect comparisons for certain treatments, which may diminish the strength of the evidence presented. Consequently, there is a pressing need for more rigorous, high-quality randomized controlled trials to further validate the efficacy and safety of natural extracts in the treatment of breast cancer.

## Data Availability

The original contributions presented in the study are included in the article/Supplementary Material, further inquiries can be directed to the corresponding authors.
