# Supplementary material for: Systematic review and network meta-analysis of the effects of plant active substance on quality of life in breast cancer patients
Source: Front Pharmacol. 2025 Nov 28;16:1622479. doi: 10.3389/fphar.2025.1622479 (PMC12698561; doi:10.3389/fphar.2025.1622479)
Supplement: Supplementary file 1 [file Table1.docx]

**Table S1.** Node-Splitting Method of Quality of Life.

| Direct | | Indirect | | Difference | |  | tau |
| --- | --- | --- | --- | --- | --- | --- | --- |
| Coef. Std. Err. | | Coef. Std. Err. | | Coef. Std. Err. | | P>\|z\| |  |
| 0.0480105 | 0.5060387 | -0.8588996 | 100.1983 | 0.9069101 | 100.1996 | 0.993 | 0.4615697 |
| 0.607743 | 0.6303944 | -0.5110229 | 632.8834 | 1.118766 | 632.884 | 0.999 | 0.4615638 |
| -3.164668 | 0.6795186 | 3.261207 | 632.9693 | -6.425875 | 632.9701 | 0.992 | 0.4615641 |
| 0.0131266 | 0.5279921 | 0.0857402 | 631.7389 | -0.0726136 | 631.7393 | 1 | 0.461564 |
| -0.0235732 | 0.480688 | 0.1248554 | 631.4231 | -0.1484286 | 631.4233 | 1 | 0.4615643 |
| -0.2640732 | 0.5651136 | 0.3611937 | 632.2668 | -0.6252669 | 632.2673 | 0.999 | 0.4615645 |
| -0.4935496 | 0.3429654 | 0.5912878 | 448.526 | -1.084837 | 448.526 | 0.998 | 0.4615586 |
| -0.200889 | 0.5537529 | 0.2994202 | 634.0654 | -0.5003093 | 634.0658 | 0.999 | 0.4615638 |
| 0.094066 | 0.5132023 | 0.0042824 | 630.1263 | 0.0897837 | 630.1265 | 1 | 0.4615643 |
| -4.661966 | 0.6059112 | 4.758956 | 631.8964 | -9.420921 | 631.8969 | 0.988 | 0.4615645 |

**Table S2.** Node-Splitting Method of fatigue.

| Side | Direct | | Indirect | | Difference | |  | tau |
| --- | --- | --- | --- | --- | --- | --- | --- | --- |
|  | Coef. Std. Err. | | Coef. Std. Err. | | Coef. Std. Err. | | P>\|z\| |  |
| A I * | 0.2761758 | 1.357535 | 0.8512609 | 105.3872 | -0.5750851 | 105.3959 | 0.996 | 1.352033 |
| B I * | 0.0738752 | 1.37609 | 0.4787186 | 632.6746 | -0.4048434 | 632.6762 | 0.999 | 1.351977 |
| C I * | 0.3115137 | 1.378696 | 0.2412771 | 632.4969 | 0.0702366 | 632.4985 | 1 | 1.351977 |
| D I * | 0.4536103 | 1.358799 | 0.0989582 | 632.639 | 0.3546521 | 632.6405 | 1 | 1.351978 |
| E I * | -0.5716806 | 0.988337 | 1.124357 | 447.1395 | -1.696038 | 447.1408 | 0.997 | 1.351985 |
| F I * | 0.4684965 | 0.9616432 | 0.0841048 | 447.3766 | 0.3843917 | 447.3777 | 0.999 | 1.351984 |
| G I * | 0.9077376 | 1.372231 | -0.3555434 | 632.3478 | 1.263281 | 632.3494 | 0.998 | 1.351978 |
| H I * | 6.121045 | 1.436843 | -5.568649 | 632.5437 | 11.68969 | 632.5457 | 0.985 | 1.351977 |

**Table S3.** Node-Splitting Method of nausea.

| Side | Direct | | Indirect | | Difference | |  | tau |
| --- | --- | --- | --- | --- | --- | --- | --- | --- |
|  | Coef. Std. Err. | | Coef. Std. Err. | | Coef. Std. Err. | | P>\|z\| |  |
| A G * | -0.7718248 | 0.3168243 | 0.5764829 | 129.2124 | -1.348308 | 129.2128 | 0.992 | 0.1921417 |
| B G * | -0.1734059 | 0.234533 | -1.368443 | 637.2398 | 1.195037 | 637.2399 | 0.999 | 0.1921419 |
| C G * | 1.032401 | 0.3020953 | -2.577581 | 629.9038 | 3.609982 | 629.9039 | 0.995 | 0.1921409 |
| D G * | 0.3146762 | 0.1728548 | -1.860153 | 456.3684 | 2.174829 | 456.3688 | 0.996 | 0.192142 |
| E G * | 0.3480723 | 0.2965132 | -1.891675 | 627.8401 | 2.239747 | 627.8403 | 0.997 | 0.192142 |
| F G * | 1.628101 | 0.3013847 | -3.176515 | 645.978 | 4.804616 | 645.9781 | 0.994 | 0.1921419 |

**Table S4.** Forest plot


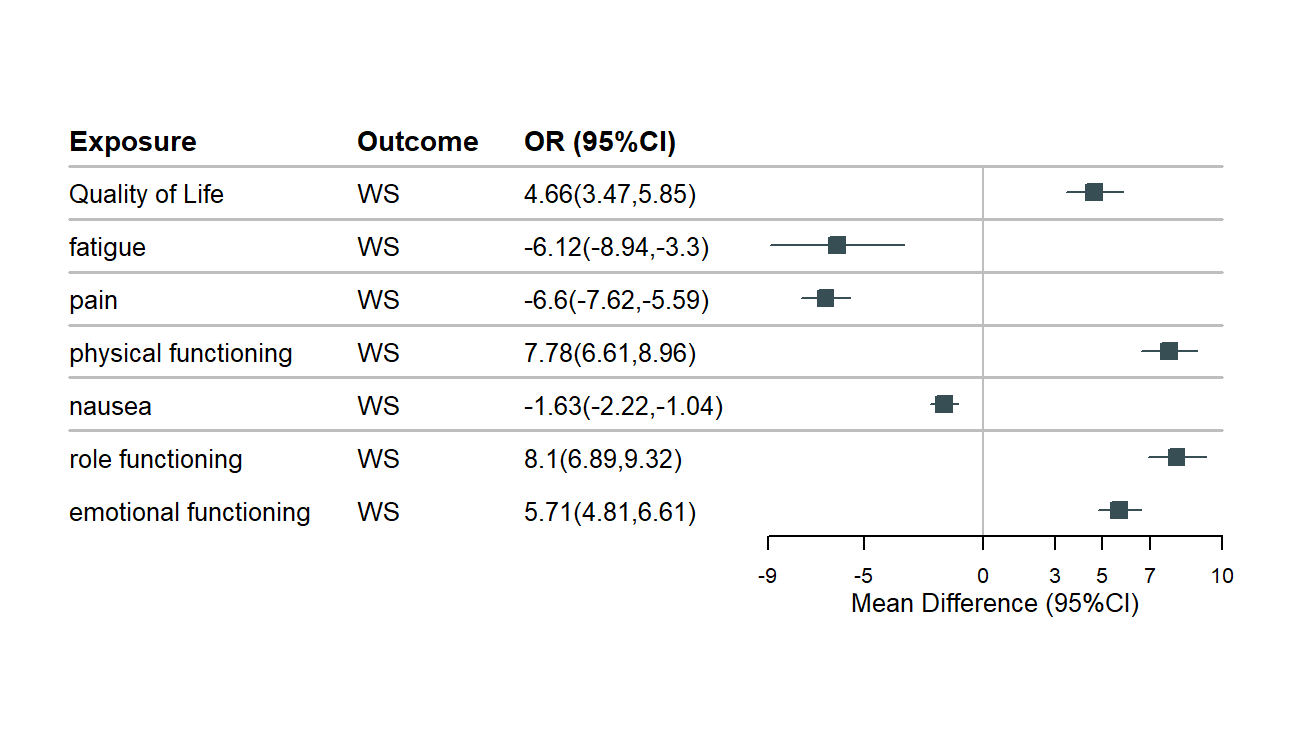


**Table S5.** League table on Quality of Life. Abbreviations: WS, Withania somnifera; VAE, Viscum album [L.] extracts; SC, soy capsules; PS76A2, an aqueous mistletoe extract standardised to the galactoside-specific mistletoe lectin; POH, P. ovate husk; MS, fermented soybean extract MicrSoy-20; HA, Helixor A; GS, ginseng; CE, Chlorella extract; CC, curcumin.

| WS | GS | PS76A2 | POH | SC | MS | HA | Standard therapy | CC | VAE | CE |
| --- | --- | --- | --- | --- | --- | --- | --- | --- | --- | --- |
| WS | -1.50 (-3.28,0.29) | -4.17 (-5.53,-2.80) | -4.40 (-6.02,-2.77) | -4.46 (-6.07,-2.85) | -4.64 (-6.15,-3.12) | -4.68 (-6.25,-3.10) | -4.66 (-5.85,-3.47) | -4.71 (-6.26,-3.16) | -4.76 (-6.31,-3.20) | -5.27 (-6.98,-3.56) |
| 1.50 (-0.29,3.28) | GS | -2.67 (-4.16,-1.18) | -2.90 (-4.63,-1.17) | -2.96 (-4.68,-1.25) | -3.14 (-4.77,-1.51) | -3.18 (-4.86,-1.49) | -3.16 (-4.50,-1.83) | -3.21 (-4.87,-1.55) | -3.26 (-4.93,-1.59) | -3.77 (-5.59,-1.96) |
| **4.17 (2.80,5.53)** | **2.67 (1.18,4.16)** | PS76A2 | -0.23 (-1.53,1.07) | -0.29 (-1.57,0.98) | -0.47 (-1.63,0.69) | -0.51 (-1.74,0.73) | -0.49 (-1.17,0.18) | -0.54 (-1.74,0.66) | -0.59 (-1.80,0.62) | -1.10 (-2.51,0.31) |
| **4.40 (2.77,6.02)** | **2.90 (1.17,4.63)** | 0.23 (-1.07,1.53) | POH | -0.06 (-1.61,1.49) | -0.24 (-1.69,1.21) | -0.28 (-1.79,1.24) | -0.26 (-1.37,0.84) | -0.31 (-1.80,1.17) | -0.36 (-1.85,1.14) | -0.87 (-2.53,0.79) |
| **4.46 (2.85,6.07)** | **2.96 (1.25,4.68)** | 0.29 (-0.98,1.57) | 0.06 (-1.49,1.61) | SC | -0.18 (-1.61,1.26) | -0.21 (-1.71,1.29) | -0.20 (-1.29,0.88) | -0.25 (-1.72,1.22) | -0.29 (-1.77,1.18) | -0.81 (-2.45,0.84) |
| **4.64 (3.12,6.15)** | **3.14 (1.51,4.77)** | 0.47 (-0.69,1.63) | 0.24 (-1.21,1.69) | 0.18 (-1.26,1.61) | MS | -0.04 (-1.44,1.36) | -0.02 (-0.97,0.92) | -0.07 (-1.44,1.30) | -0.12 (-1.50,1.26) | -0.63 (-2.19,0.92) |
| **4.68 (3.10,6.25)** | **3.18 (1.49,4.86)** | 0.51 (-0.73,1.74) | 0.28 (-1.24,1.79) | 0.21 (-1.29,1.71) | 0.04 (-1.36,1.44) | HA | 0.01 (-1.02,1.05) | -0.03 (-1.47,1.40) | -0.08 (-1.52,1.36) | -0.59 (-2.21,1.02) |
| **4.66 (3.47,5.85)** | **3.16 (1.83,4.50)** | 0.49 (-0.18,1.17) | 0.26 (-0.84,1.37) | 0.20 (-0.88,1.29) | 0.02 (-0.92,0.97) | -0.01 (-1.05,1.02) | standard therapy | -0.05 (-1.04,0.94) | -0.09 (-1.10,0.91) | -0.61 (-1.84,0.63) |
| **4.71 (3.16,6.26)** | **3.21 (1.55,4.87)** | 0.54 (-0.66,1.74) | 0.31 (-1.17,1.80) | 0.25 (-1.22,1.72) | 0.07 (-1.30,1.44) | 0.03 (-1.40,1.47) | 0.05 (-0.94,1.04) | CC | -0.05 (-1.46,1.37) | -0.56 (-2.14,1.02) |
| **4.76 (3.20,6.31)** | **3.26 (1.59,4.93)** | 0.59 (-0.62,1.80) | 0.36 (-1.14,1.85) | 0.29 (-1.18,1.77) | 0.12 (-1.26,1.50) | 0.08 (-1.36,1.52) | 0.09 (-0.91,1.10) | 0.05 (-1.37,1.46) | VAE | -0.51 (-2.11,1.08) |
| **5.27 (3.56,6.98)** | **3.77 (1.96,5.59)** | 1.10 (-0.31,2.51) | 0.87 (-0.79,2.53) | 0.81 (-0.84,2.45) | 0.63 (-0.92,2.19) | 0.59 (-1.02,2.21) | 0.61 (-0.63,1.84) | 0.56 (-1.02,2.14) | 0.51 (-1.08,2.11) | CE |

**Table S6.** League table on fatigue. Abbreviations: WS, Withania somnifera; VAE, Viscum album [L.] extracts; PS76A2, an aqueous mistletoe extract standardised to the galactoside-specific mistletoe lectin; PC, Paullinia cupana; MS-20, fermented soybean extract MicrSoy-20; JA, Jollab; HA, Helixor A; GS, ginseng.

| WS | VAE | PS76A2 | MS-20 | JA | GS | HA | standard therapy | PC |
| --- | --- | --- | --- | --- | --- | --- | --- | --- |
| WS | 5.21 (1.32,9.11) | 5.65 (2.26,9.04) | 5.67 (1.79,9.54) | 5.81 (1.91,9.71) | 5.84 (1.97,9.72) | 6.05 (2.15,9.95) | 6.12 (3.30,8.94) | 6.69 (3.27,10.11) |
| **-5.21 (-9.11,-1.32)** | VAE | 0.44 (-2.84,3.72) | 0.45 (-3.33,4.24) | 0.60 (-3.22,4.41) | 0.63 (-3.15,4.41) | 0.83 (-2.98,4.64) | 0.91 (-1.78,3.60) | 1.48 (-1.84,4.79) |
| **-5.65 (-9.04,-2.26)** | -0.44 (-3.72,2.84) | PS76A2 | 0.01 (-3.25,3.28) | 0.16 (-3.14,3.45) | 0.19 (-3.07,3.45) | 0.39 (-2.90,3.68) | 0.47 (-1.42,2.35) | 1.04 (-1.66,3.74) |
| **-5.67 (-9.54,-1.79)** | -0.45 (-4.24,3.33) | -0.01 (-3.28,3.25) | MS-20 | 0.14 (-3.65,3.94) | 0.18 (-3.59,3.94) | 0.38 (-3.41,4.17) | 0.45 (-2.21,3.12) | 1.03 (-2.27,4.32) |
| **-5.81 (-9.71,-1.91)** | -0.60 (-4.41,3.22) | -0.16 (-3.45,3.14) | -0.14 (-3.94,3.65) | JA | 0.04 (-3.76,3.83) | 0.24 (-3.58,4.06) | 0.31 (-2.39,3.01) | 0.88 (-2.44,4.21) |
| **-5.84 (-9.72,-1.97)** | -0.63 (-4.41,3.15) | -0.19 (-3.45,3.07) | -0.18 (-3.94,3.59) | -0.04 (-3.83,3.76) | GS | 0.20 (-3.59,3.99) | 0.28 (-2.38,2.94) | 0.85 (-2.44,4.14) |
| **-6.05 (-9.95,-2.15)** | -0.83 (-4.64,2.98) | -0.39 (-3.68,2.90) | -0.38 (-4.17,3.41) | -0.24 (-4.06,3.58) | -0.20 (-3.99,3.59) | HA | 0.07 (-2.62,2.77) | 0.65 (-2.68,3.97) |
| **-6.12 (-8.94,-3.30)** | -0.91 (-3.60,1.78) | -0.47 (-2.35,1.42) | -0.45 (-3.12,2.21) | -0.31 (-3.01,2.39) | -0.28 (-2.94,2.38) | -0.07 (-2.77,2.62) | standard therapy | 0.57 (-1.37,2.51) |
| **-6.69 (-10.11,-3.27)** | -1.48 (-4.79,1.84) | -1.04 (-3.74,1.66) | -1.03 (-4.32,2.27) | -0.88 (-4.21,2.44) | -0.85 (-4.14,2.44) | -0.65 (-3.97,2.68) | -0.57 (-2.51,1.37) | PC |

**Table S7.** League table on pain. Abbreviations: WS, Withania somnifera; VAE, Viscum album [L.] extracts; RB, robuvit®( a natural supplement extracted from the French oak wood); PS76A2, an aqueous mistletoe extract standardised to the galactoside-specific mistletoe lectin; POH, P. ovate husk; MS-20, fermented soybean extract MicrSoy-20; HA, Helixor A; CE, Chlorella extract;

| WS | RB | VAE | POH | HA | CE | MS-20 | PS76A2 | standard therapy |
| --- | --- | --- | --- | --- | --- | --- | --- | --- |
| WS | 5.12 (3.96,6.28) | 5.69 (4.58,6.81) | 5.94 (4.73,7.15) | 6.21 (5.08,7.35) | 6.60 (5.31,7.90) | 6.57 (5.52,7.62) | 6.58 (5.54,7.62) | 6.60 (5.59,7.62) |
| **-5.12 (-6.28,-3.96)** | RB | 0.57 (-0.15,1.29) | 0.82 (-0.04,1.68) | 1.09 (0.34,1.84) | 1.48 (0.51,2.46) | 1.45 (0.84,2.06) | 1.46 (0.87,2.05) | 1.48 (0.93,2.04) |
| **-5.69 (-6.81,-4.58)** | -0.57 (-1.29,0.15) | VAE | 0.25 (-0.56,1.05) | 0.52 (-0.17,1.20) | 0.91 (-0.02,1.83) | 0.88 (0.35,1.41) | 0.88 (0.38,1.39) | 0.91 (0.45,1.37) |
| **-5.94 (-7.15,-4.73)** | -0.82 (-1.68,0.04) | -0.25 (-1.05,0.56) | POH | 0.27 (-0.56,1.10) | 0.66 (-0.38,1.70) | 0.63 (-0.08,1.34) | 0.64 (-0.05,1.33) | 0.66 (0.01,1.32) |
| **-6.21 (-7.35,-5.08)** | **-1.09 (-1.84,-0.34)** | -0.52 (-1.20,0.17) | -0.27 (-1.10,0.56) | HA | 0.39 (-0.56,1.34) | 0.36 (-0.21,0.93) | 0.37 (-0.18,0.92) | 0.39 (-0.12,0.90) |
| **-6.60 (-7.90,-5.31)** | **-1.48 (-2.46,-0.51)** | -0.91 (-1.83,0.02) | -0.66 (-1.70,0.38) | -0.39 (-1.34,0.56) | CE | -0.03 (-0.88,0.81) | -0.02 (-0.85,0.81) | 0.00 (-0.80,0.80) |
| **-6.57 (-7.62,-5.52)** | **-1.45 (-2.06,-0.84)** | **-0.88 (-1.41,-0.35)** | -0.63 (-1.34,0.08) | -0.36 (-0.93,0.21) | 0.03 (-0.81,0.88) | MS-20 | 0.01 (-0.33,0.35) | 0.03 (-0.23,0.29) |
| **-6.58 (-7.62,-5.54)** | **-1.46 (-2.05,-0.87)** | **-0.88 (-1.39,-0.38)** | -0.64 (-1.33,0.05) | -0.37 (-0.92,0.18) | 0.02 (-0.81,0.85) | -0.01 (-0.35,0.33) | PS76A2 | 0.02 (-0.19,0.24) |
| **-6.60 (-7.62,-5.59)** | **-1.48 (-2.04,-0.93)** | **-0.91 (-1.37,-0.45)** | **-0.66 (-1.32,-0.01)** | -0.39 (-0.90,0.12) | -0.00 (-0.80,0.80) | -0.03 (-0.29,0.23) | -0.02 (-0.24,0.19) | standard therapy |

**Table S8.** League table on physical functioning. Abbreviations: WS, Withania somnifera; VAE, Viscum album [L.] extracts; PS76A2, an aqueous mistletoe extract standardised to the galactoside-specific mistletoe lectin; MS-20, fermented soybean extract MicrSoy-20; HA, Helixor A; GS, ginseng; CE, Chlorella extract; CC, curcumin.

| WS | GS | CC | PS76A2 | MS-20 | VAE | HA | standard therapy | CE |
| --- | --- | --- | --- | --- | --- | --- | --- | --- |
| WS | -5.30 (-6.75,-3.84) | -6.80 (-8.05,-5.55) | -7.08 (-8.28,-5.89) | -7.50 (-8.70,-6.29) | -7.51 (-8.76,-6.25) | -7.74 (-9.02,-6.47) | -7.78 (-8.96,-6.61) | -8.30 (-9.73,-6.87) |
| **5.30 (3.84,6.75)** | GS | -1.50 (-2.47,-0.53) | -1.79 (-2.68,-0.89) | -2.20 (-3.10,-1.30) | -2.21 (-3.18,-1.24) | -2.45 (-3.45,-1.45) | -2.49 (-3.35,-1.62) | -3.00 (-4.19,-1.82) |
| **6.80 (5.55,8.05)** | **1.50 (0.53,2.47)** | CC | -0.29 (-0.77,0.20) | -0.70 (-1.21,-0.19) | -0.71 (-1.33,-0.09) | -0.95 (-1.61,-0.28) | -0.99 (-1.42,-0.56) | -1.50 (-2.43,-0.58) |
| **7.08 (5.89,8.28)** | **1.79 (0.89,2.68)** | 0.29 (-0.20,0.77) | PS76A2 | -0.41 (-0.76,-0.07) | -0.42 (-0.92,0.07) | -0.66 (-1.21,-0.11) | -0.70 (-0.92,-0.48) | -1.22 (-2.06,-0.37) |
| **7.50 (6.29,8.70)** | **2.20 (1.30,3.10)** | **0.70 (0.19,1.21)** | **0.41 (0.07,0.76)** | MS-20 | -0.01 (-0.53,0.50) | -0.25 (-0.81,0.32) | -0.29 (-0.55,-0.02) | -0.80 (-1.66,0.05) |
| **7.51 (6.25,8.76)** | **2.21 (1.24,3.18)** | **0.71 (0.09,1.33)** | 0.42 (-0.07,0.92) | 0.01 (-0.50,0.53) | VAE | -0.24 (-0.91,0.43) | -0.28 (-0.72,0.16) | -0.79 (-1.72,0.13) |
| **7.74 (6.47,9.02)** | **2.45 (1.45,3.45)** | **0.95 (0.28,1.61)** | **0.66 (0.11,1.21)** | 0.25 (-0.32,0.81) | 0.24 (-0.43,0.91) | HA | -0.04 (-0.54,0.46) | -0.56 (-1.52,0.40) |
| **7.78 (6.61,8.96)** | **2.49 (1.62,3.35)** | **0.99 (0.56,1.42)** | **0.70 (0.48,0.92)** | **0.29 (0.02,0.55)** | 0.28 (-0.16,0.72) | 0.04 (-0.46,0.54) | standard therapy | -0.52 (-1.33,0.30) |
| **8.30 (6.87,9.73)** | **3.00 (1.82,4.19)** | **1.50 (0.58,2.43)** | **1.22 (0.37,2.06)** | 0.80 (-0.05,1.66) | 0.79 (-0.13,1.72) | 0.56 (-0.40,1.52) | 0.52 (-0.30,1.33) | CE |

**Table S9.** League table on nausea. Abbreviations: WS, Withania somnifera; VAE, Viscum album [L.] extracts; PS76A2, an aqueous mistletoe extract standardised to the galactoside-specific mistletoe lectin; PEP, Peppermint; MS-20, fermented soybean extract MicrSoy-20; GI, ginger.

| WS | PEP | PS76A2 | VAE | standard therapy | MS-20 | GI |
| --- | --- | --- | --- | --- | --- | --- |
| WS | 0.60 (-0.24,1.43) | 1.31 (0.63,1.99) | 1.28 (0.45,2.11) | 1.63 (1.04,2.22) | 1.80 (1.05,2.55) | 2.40 (1.54,3.26) |
| **-0.60 (-1.43,0.24)** | PEP | 0.72 (0.04,1.40) | 0.68 (-0.15,1.51) | 1.03 (0.44,1.62) | 1.21 (0.46,1.96) | 1.80 (0.95,2.66) |
| **-1.31 (-1.99,-0.63)** | **-0.72 (-1.40,-0.04)** | PS76A2 | -0.03 (-0.71,0.64) | 0.31 (-0.02,0.65) | 0.49 (-0.08,1.06) | 1.09 (0.38,1.79) |
| **-1.28 (-2.11,-0.45)** | -0.68 (-1.51,0.15) | 0.03 (-0.64,0.71) | VAE | 0.35 (-0.23,0.93) | 0.52 (-0.22,1.26) | 1.12 (0.27,1.97) |
| **-1.63 (-2.22,-1.04)** | **-1.03 (-1.62,-0.44)** | -0.31 (-0.65,0.02) | -0.35 (-0.93,0.23) | standard therapy | 0.17 (-0.29,0.63) | 0.77 (0.15,1.39) |
| **-1.80 (-2.55,-1.05)** | **-1.21 (-1.96,-0.46)** | -0.49 (-1.06,0.08) | -0.52 (-1.26,0.22) | -0.17 (-0.63,0.29) | MS-20 | 0.60 (-0.17,1.37) |
| **-2.40 (-3.26,-1.54)** | **-1.80 (-2.66,-0.95)** | **-1.09 (-1.79,-0.38)** | **-1.12 (-1.97,-0.27)** | **-0.77 (-1.39,-0.15)** | -0.60 (-1.37,0.17) | GI |

**Table S10.** League table on role functioning. Abbreviations: WS, Withania somnifera; VAE, Viscum album [L.] extracts; PS76A2, an aqueous mistletoe extract standardised to the galactoside-specific mistletoe lectin; MS-20, fermented soybean extract MicrSoy-20; HA, Helixor A; GS, ginseng; CE, Chlorella extract.

| WS | GS | VAE | PS76A2 | HA | MS-20 | CE | standard therapy |
| --- | --- | --- | --- | --- | --- | --- | --- |
| WS | -5.42 (-6.93,-3.92) | -7.20 (-8.50,-5.90) | -7.60 (-8.84,-6.37) | -7.71 (-9.03,-6.40) | -7.92 (-9.17,-6.68) | -8.10 (-9.57,-6.64) | -8.10 (-9.32,-6.89) |
| **5.42 (3.92,6.93)** | GS | -1.77 (-2.78,-0.77) | -2.18 (-3.10,-1.26) | -2.29 (-3.32,-1.26) | -2.50 (-3.43,-1.56) | -2.68 (-3.89,-1.47) | -2.68 (-3.57,-1.78) |
| **7.20 (5.90,8.50)** | **1.77 (0.77,2.78)** | VAE | -0.40 (-0.91,0.11) | -0.52 (-1.20,0.17) | -0.72 (-1.25,-0.19) | -0.91 (-1.85,0.03) | -0.91 (-1.37,-0.45) |
| **7.60 (6.37,8.84)** | **2.18 (1.26,3.10)** | 0.40 (-0.11,0.91) | PS76A2 | -0.11 (-0.66,0.44) | -0.32 (-0.66,0.02) | -0.50 (-1.35,0.34) | -0.50 (-0.72,-0.29) |
| **7.71 (6.40,9.03)** | **2.29 (1.26,3.32)** | 0.52 (-0.17,1.20) | 0.11 (-0.44,0.66) | HA | -0.21 (-0.78,0.36) | -0.39 (-1.35,0.57) | -0.39 (-0.90,0.12) |
| **7.92 (6.68,9.17)** | **2.50 (1.56,3.43)** | **0.72 (0.19,1.25)** | 0.32 (-0.02,0.66) | 0.21 (-0.36,0.78) | MS-20 | -0.18 (-1.04,0.68) | -0.18 (-0.45,0.08) |
| **8.10 (6.64,9.57)** | **2.68 (1.47,3.89)** | 0.91 (-0.03,1.85) | 0.50 (-0.34,1.35) | 0.39 (-0.57,1.35) | 0.18 (-0.68,1.04) | CE | -0.00 (-0.82,0.82) |
| **8.10 (6.89,9.32)** | **2.68 (1.78,3.57)** | **0.91 (0.45,1.37)** | **0.50 (0.29,0.72)** | 0.39 (-0.12,0.90) | 0.18 (-0.08,0.45) | 0.00 (-0.82,0.82) | standard therapy |

**Table S11.** League table on emotional functioning. Abbreviations: WS, Withania somnifera; VAE, Viscum album [L.] extracts; PS76A2, an aqueous mistletoe extract standardised to the galactoside-specific mistletoe lectin; MS-20, fermented soybean extract MicrSoy-20; HA, Helixor A; GS, ginseng; CE, Chlorella extract.

| WS | GS | PS76A2 | VAE | HA | standard therapy | MS-20 | CE |
| --- | --- | --- | --- | --- | --- | --- | --- |
| WS | -3.01 (-4.28,-1.74) | -5.19 (-6.12,-4.27) | -5.19 (-6.20,-4.19) | -5.58 (-6.61,-4.55) | -5.71 (-6.61,-4.81) | -5.80 (-6.74,-4.87) | -6.24 (-7.47,-5.01) |
| **3.01 (1.74,4.28)** | GS | -2.18 (-3.11,-1.26) | -2.18 (-3.19,-1.18) | -2.57 (-3.60,-1.54) | -2.70 (-3.60,-1.80) | -2.79 (-3.73,-1.86) | -3.23 (-4.46,-2.00) |
| **5.19 (4.27,6.12)** | **2.18 (1.26,3.11)** | PS76A2 | -0.00 (-0.50,0.49) | -0.39 (-0.94,0.16) | -0.52 (-0.74,-0.30) | -0.61 (-0.95,-0.27) | -1.05 (-1.91,-0.19) |
| **5.19 (4.19,6.20)** | **2.18 (1.18,3.19)** | 0.00 (-0.49,0.50) | VAE | -0.39 (-1.06,0.28) | -0.52 (-0.96,-0.07) | -0.61 (-1.13,-0.09) | -1.05 (-1.99,-0.10) |
| **5.58 (4.55,6.61)** | **2.57 (1.54,3.60)** | 0.39 (-0.16,0.94) | 0.39 (-0.28,1.06) | HA | -0.13 (-0.63,0.38) | -0.22 (-0.79,0.35) | -0.66 (-1.63,0.32) |
| **5.71 (4.81,6.61)** | **2.70 (1.80,3.60)** | **0.52 (0.30,0.74)** | **0.52 (0.07,0.96)** | 0.13 (-0.38,0.63) | standard therapy | -0.09 (-0.36,0.17) | -0.53 (-1.37,0.30) |
| **5.80 (4.87,6.74)** | **2.79 (1.86,3.73)** | **0.61 (0.27,0.95)** | **0.61 (0.09,1.13)** | 0.22 (-0.35,0.79) | 0.09 (-0.17,0.36) | MS-20 | -0.44 (-1.31,0.44) |
| **6.24 (5.01,7.47)** | **3.23 (2.00,4.46)** | **1.05 (0.19,1.91)** | **1.05 (0.10,1.99)** | 0.66 (-0.32,1.63) | 0.53 (-0.30,1.37) | 0.44 (-0.44,1.31) | CE |

**Table S12.** List adverse events.

| Author | Year | Treatment | nausea | Injection and infusion  site reactions | Conjunctivitis | Febrile temperature | Sting | oral intolerance | gastritis | flatulence |
| --- | --- | --- | --- | --- | --- | --- | --- | --- | --- | --- |
| Hasheminasab et al. | 2020 | P. ovate husk | 2/21 |  |  |  |  |  |  |  |
| Pelzer et al. | 2018 | Viscum album [L.] extracts |  | 1/64 |  |  |  |  |  |  |
| Troeger et al. | 2014 | Helixor A |  | 20/34 | 1/34 | 1/34 | 1/34 |  |  |  |
| Biswal et al. | 2012 | Withania somnifera |  |  |  |  |  | 1/50 | 1/50 | 1/50 |
| Semiglazov et al. | 2006 | Standardised Mistletoe Extract PS76A2 |  | 31/176 |  |  |  |  |  |  |
